# Use of sildenafil and l-arginine in an experimental rat model for the prevention of neonatal necrotizing enterocolitis

**DOI:** 10.1038/s41598-022-10323-8

**Published:** 2022-04-13

**Authors:** Gabriela Araujo Moreira, André Ivan Bradley dos Santos Dias, Silvia Maria Suter Correia Cadena, Marília Locatelli Corrêa-Ferreira, Sergio Ossamu Ioshii, Camila Girardi Fachin

**Affiliations:** 1 Pediatric Surgery Department, Medical School, Federal University of Parana, Curitiba, R. Gen. Carneiro, 181 - Alto da Glória, Curitiba, PR 80060-900 Brazil; 2 Pediatric Surgery Department, Federal University of Parana, Curitiba, R. Gen. Carneiro, 181 - Alto da Glória, Curitiba, PR 80060-900 Brazil; 3 Biochemistry Department, Federal University of Parana Campus Polytechnic Center, Curitiba, Jardim das Américas, Curitiba, PR 80050-540 Brazil; 4 Pathology Department, Federal University of Parana, Curitiba, R. Gen. Carneiro, 181 - Alto da Glória, Curitiba, PR 80060-900 Brazil

**Keywords:** Medical research, Gastrointestinal diseases

## Abstract

Necrotizing enterocolitis (NEC) has a 45% mortality in neonatal intensive care units. This paper aimed to evaluate the isolated and combined effects of sildenafil and l-arginine in the prevention of necrotizing enterocolitis. Neonatal rats were fed formula milk and submitted to hypoxia under a 100% N2 atmosphere for 70 s. Then, animals were subjected to hypothermia (4 °C for 10 min), twice a day for 3 days. Forty neonatal rats were divided into five groups: negative control—not submitted to the protocol (n = 5), sildenafil group—NEC protocol (n = 9), l-arginine group—NEC protocol (n = 9), l-arginine and sildenafil group—NEC protocol (n = 9) and positive control—NEC protocol and intraperitoneal saline solution (n = 8). Jejunum and terminal ileus were removed for histopathologic and immunohistochemical Ki-67 analysis. Kruskal–Wallis test was used to analyze mortality, survival, body weight, intestinal injury score and Ki-67 proliferation index. All animals submitted to the protocol developed enterocolitis. Mortality rate was higher in group that received only l-arginine (p = 0.0293). The Ki-67 analysis showed a higher proliferative index in groups that received interventional drugs (p = 0.017). In conclusion, sildenafil and l-arginine were not effective to reduce intestinal injury.

## Introduction

Neonatal necrotizing enterocolitis (NEC) is an intestinal inflammatory disorder. It affects approximately 5% to 7% of premature newborns and it is one of the major causes of mortality in neonatal intensive care units (45%)^1^. Prematurity is the main risk factor for the disease, and more than 90% of newborns affected by NEC are born at less than 37 weeks of gestational age^2^.

Multiple factors are involved in the pathophysiology of the disease, including the immaturity of the immune system to the complex changing composition of the intestinal microbiota. In this regard, perinatal hypoxia, early feeding with formula milk, and bacterial colonization play a fundamental role in the activation of the inflammatory cascade of NEC. These factors induce intestinal damage, which may progress to coagulative necrosis of the gastrointestinal tract^1,3^.

Premature bowel microcirculatory perfusion is mainly regulated by the vasodilator nitric oxide (NO), which is generated through endothelial nitric-oxide synthase (eNOS) activity^4^. When harmful bacteria reach the circulation, they inhibit the expression of the endothelial nitric-oxide synthase (eNOS). Consequently, this process reduces the availability of nitric oxide (NO), an important modulator of homeostatic and inflammatory conditions^1^. The reduction in plasma NO levels promotes intense vasoconstriction, changes intestinal perfusion, and generates hypoxia, evolving to the characteristic necrosis of NEC^4,5^.

To understand the underlying biological mechanisms that lead to NEC, the innate immune receptor toll-like receptor 4 (TLR4) expressed on intestinal endothelial cells has been identified^6^. TLR4-deficient mice showed reduced mucosal inflammation and reduced intestinal necrosis in experimental NEC^7^. In order to test TLR4 signaling within the endothelium with reduced blood flow and impaired eNOS function, Yazji et al.^4^ evaluated the supplementation of the phosphodiesterase-5 inhibitor, sildenafil, in wild-type mice and observed a reduction in the severity of NEC, through vasodilation and maintenance of the intraluminal NO activity promoted by the substance^4^.

In this context, the administration of sildenafil, a substance that increases eNOS signaling, emerges as a possible preventive strategy for the development of NEC. Sildenafil is a robust selective inhibitor of Phosphodiesterase-5 (PDE5) which is responsible for the degradation of cyclic guanosine monophosphate (cGMP). The accumulation of cGMP leads to the activation of the NO/cGMP system, favoring the production of NO and increasing intestinal perfusion. Thus, it may prevent the appearance of necrosis^1^.

Strategies that increase nitric oxide signaling, such as providing sodium nitrate oxide precursor, or sildenafil treatment, restores mesenteric perfusion in experimental models and attenuates NEC severity^1,4^. These findings also provide insights into the protective mechanisms of breast milk, which were found especially when enriched with precursor molecules of nitric oxide as well as human milk oligosaccharides, both of which increase intestinal perfusion and prevent the deleterious effects of the bacterial signal. Intestinal exaggeration in the premature host in the pathogenesis of NEC^1,4^.

To enhance the activation effect of eNOS, we included an l-arginine supplementation. This amino acid, the precursor of NO, provides a greater amount of substrate for enzyme activity^4,5,8,9^.

It has been observed that, although the NO released in the endothelium is essential in the modulation of peripheral vascular tone, l-arginine plays an important role in endothelium-dependent relaxation, a decisive issue in the prevention of tissue damage^4,5,8,9^.

In a previous study, intraperitoneal administration of l-arginine in an experimental model of NEC showed an increase of NO in the intestinal tissue, which resulted in a lower degree of injuries produced by hypoxia and hypothermia events^10^.

The insufficient endogenous arginine synthesis could limit NO production and impair vasodilation in the postprandial intestine. Chen et al.^11^ found that supplementing arginine increases postprandial hyperaemia and prevents experimental NEC. Arginine supplementation can increase the blood flow of the intestinal microvasculature and prevent NEC, while an arginine antagonist may exacerbate NEC, suggesting that modulation of intestinal oxygen demand and supply in the premature intestine is a NEC prevention strategy^11^.

Given that NEC is an aggressive inflammatory process, we set out to study the effects of sildenafil and l-arginine—both isolated and combined—on intestinal injuries in an experimental model of necrotizing enterocolitis.

## Results

**Figure 1:**
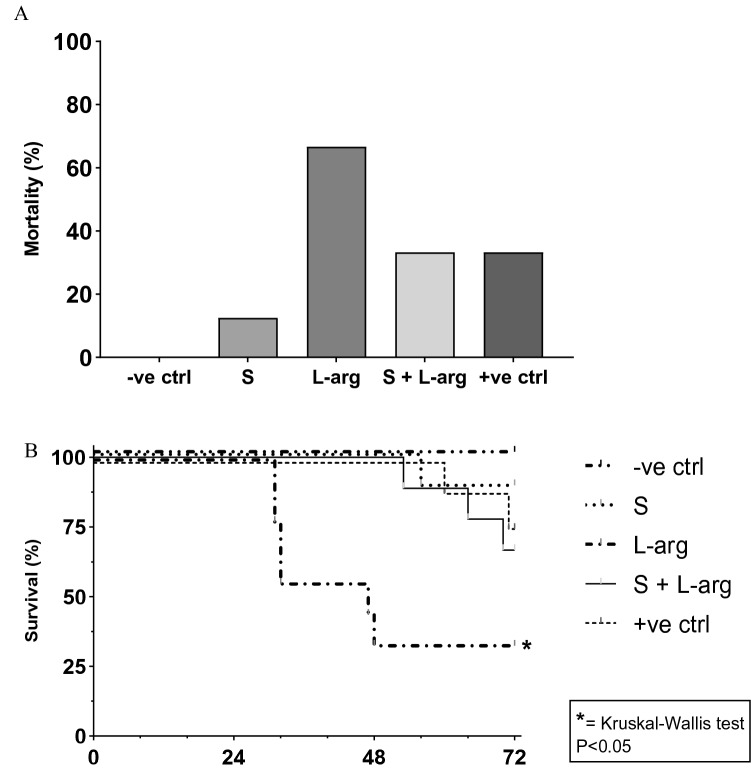
Mortality and survival. (**A**) Percentage of deaths per group over total deaths. (**B**) Kaplan–Meier curves with the mean lifespan per group (h), from the beginning to the end of the experiment. Groups: A—negative control (− ve ctrl), B—sildenafil (S), C—l-arginine (l-arg), D—sildenafil and l-arginine (S + l-arg) and E—positive control (+ ve ctrl) (Kruskal–Wallis, p = 0.0293).

After the NEC protocol, the mortality rate was 14/40 (35%). Group C, which only received l-arginine, obtained the highest mortality rate among the groups (66.67%), as shown in Fig. 1 (Kruskal–Wallis, p = 0.0293). Contrarily, group B, which only received sildenafil, obtained the lowest mortality rate among the groups submitted to the NEC protocol (14%) (Kruskal–Wallis, p = 0.0293).

Among newborn rats that died, group E had the highest life span (with a mean of 63. 83 hours of life since the beginning of the experiment) as shown in Fig. 1. Group C, on the other hand, demonstrated the lowest life span among groups (with a mean of 37.3 h of life) (Kruskal–Wallis, p = 0.0293). Among the groups that received the studied substances (sildenafil and/or l-arginine), group D had the highest life span (with a mean of 62.25 hours of life) (Kruskal–Wallis, p = 0.0293). One animal in group E was excluded from the protocol due to esophageal perforation (2.5%). As a result, a total of 25 animals were examined in this study.

**Figure 2:**
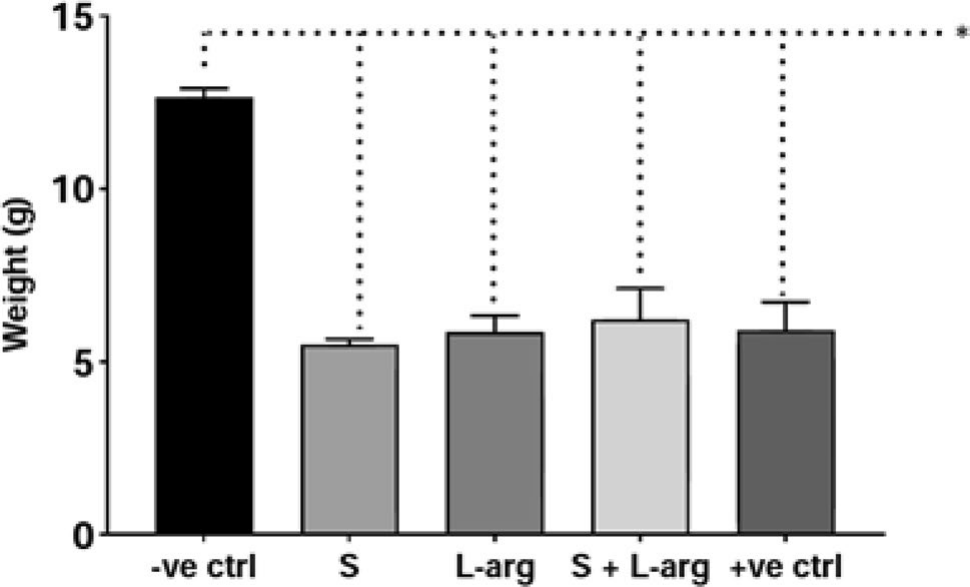
Mean body weight of the groups at the end of the experiment. Groups: A—negative control (− ve ctrl), B—sildenafil (S), C—l-arginine (l-arg), D—sildenafil and l-arginine (S + l-arg) and E—positive control (+ ve ctrl) (Kruskal–Wallis, p = 0.0088).

During the experiment, groups submitted to the NEC protocol showed decreased body weight gain, which is summarized in Fig. 2. The final bodyweight of the rats in group A obtained a mean of 12.66 g. This weight was significantly higher than all NEC groups, whose means were of 5.31 g in group B, 5.87 g in group C, 6.23 g in group D, and 5.92 g in group E (Kruskal–Wallis, p = 0.0088).

**Figure 3:**
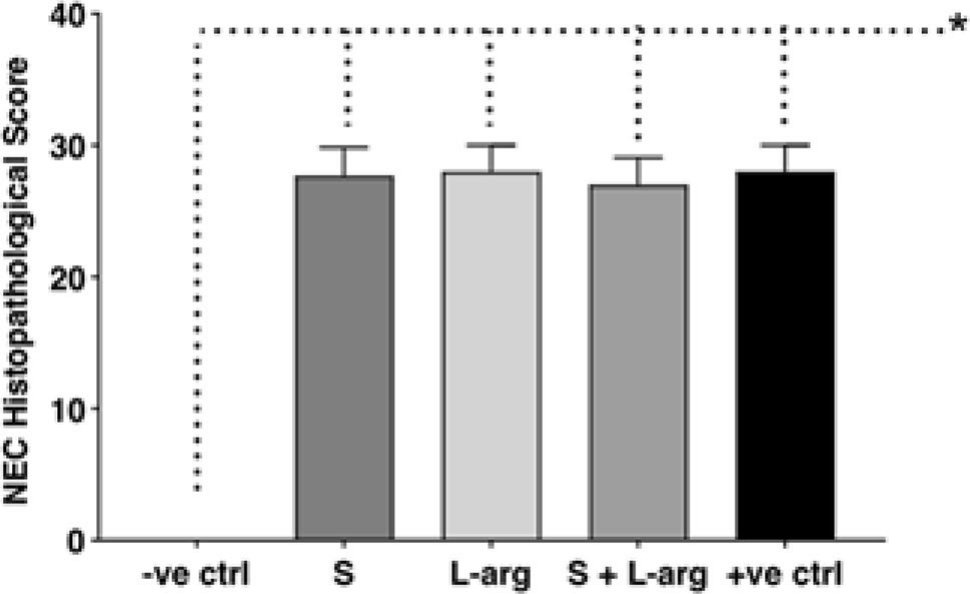
Mean histopathological injury score of the groups. Groups: A—negative control (− ve ctrl), B—sildenafil (S), C—l-arginine (l-arg), D—sildenafil and l-arginine (S + l-arg) and E—positive control (+ ve ctrl) (Kruskal–Wallis, p = 0.0288).

According to histopathological analysis, all animals submitted to the protocol developed enterocolitis (score > 18), as shown in Fig. 3. Group A presented a normal intestine with intact villi and layers. There was a significant difference in injury severity among NEC protocol groups and group A (Kruskal–Wallis, p = 0.0288). Group D had the lowest mean score for intestinal injury (26.66), although not statistically significant.

**Figure 4:**
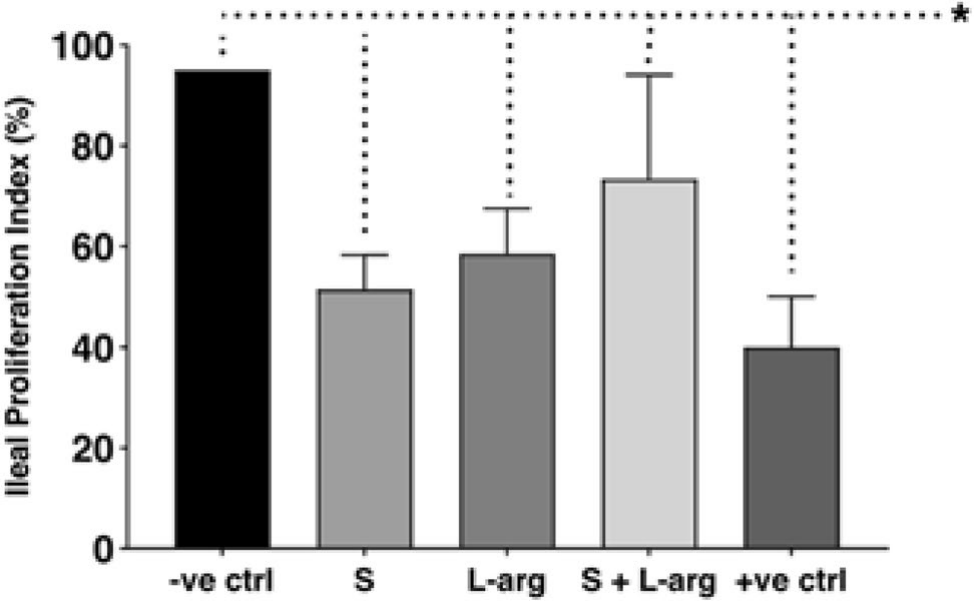
Mean proliferative index of groups. Groups: A—negative control (− ve ctrl), B—sildenafil (S), C—l-arginine (l-arg), D—sildenafil and l-arginine (S + l-arg) and E—positive control (+ ve ctrl) (Kruskal–Wallis, p = 0.0017).

The amount of metabolically active cells, measured by Ki-67, is shown in Fig. 4. A mean of 95% cell activity rate was observed in rats in group A. Group E exhibited the lowest proliferative index (a mean of 38%) (Kruskal–Wallis, (p = 0.017). Among the groups submitted to the protocol that received interventional drugs, group D (administration of sildenafil and l-arginine) had the highest rate of cellular activity (with a mean of 68.33%).

**Figure 5:**
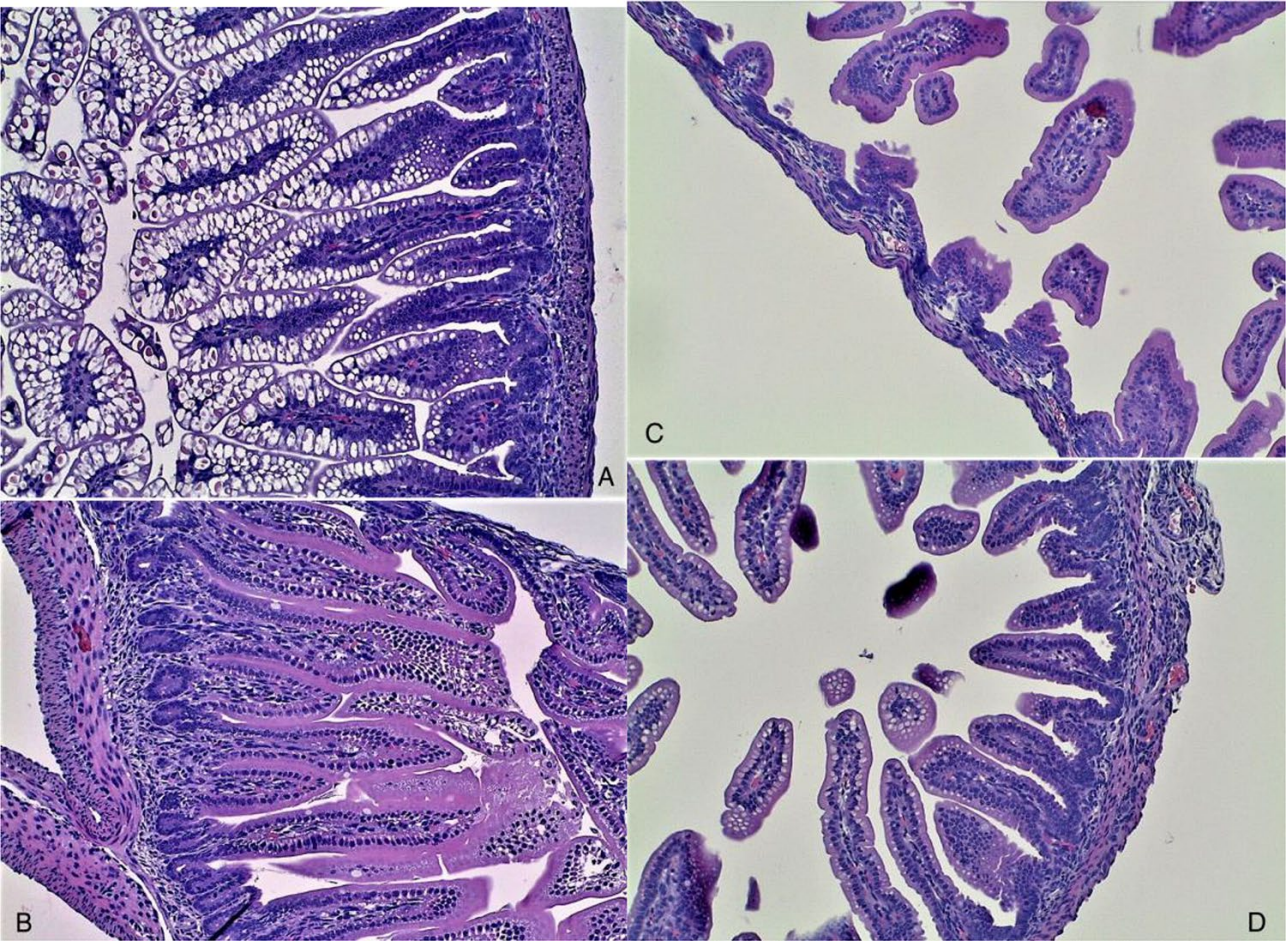
Histopathological analysis of NEC intestinal injuries. **(A)** Negative control group—normal villi: normal length and appearance. **(B)** Negative control group—Intact and preserved intestinal wall. **(C)** Group submitted to the NEC protocol—villous flattening, reduction in number of villi and thinning of the intestinal wall. **(D)** Group submitted to the NEC protocol—villous flattening and reduction in number of villi.

In the histopathological analysis of NEC intestinal injuries, the group submitted to the NEC protocol demonstrated villous flattening, reduction in the number of villi and thinning of the intestinal wall. The negative control group showed normal villi, normal length appearance and intact and preserved intestine (Fig. 5).

**Figure 6:**
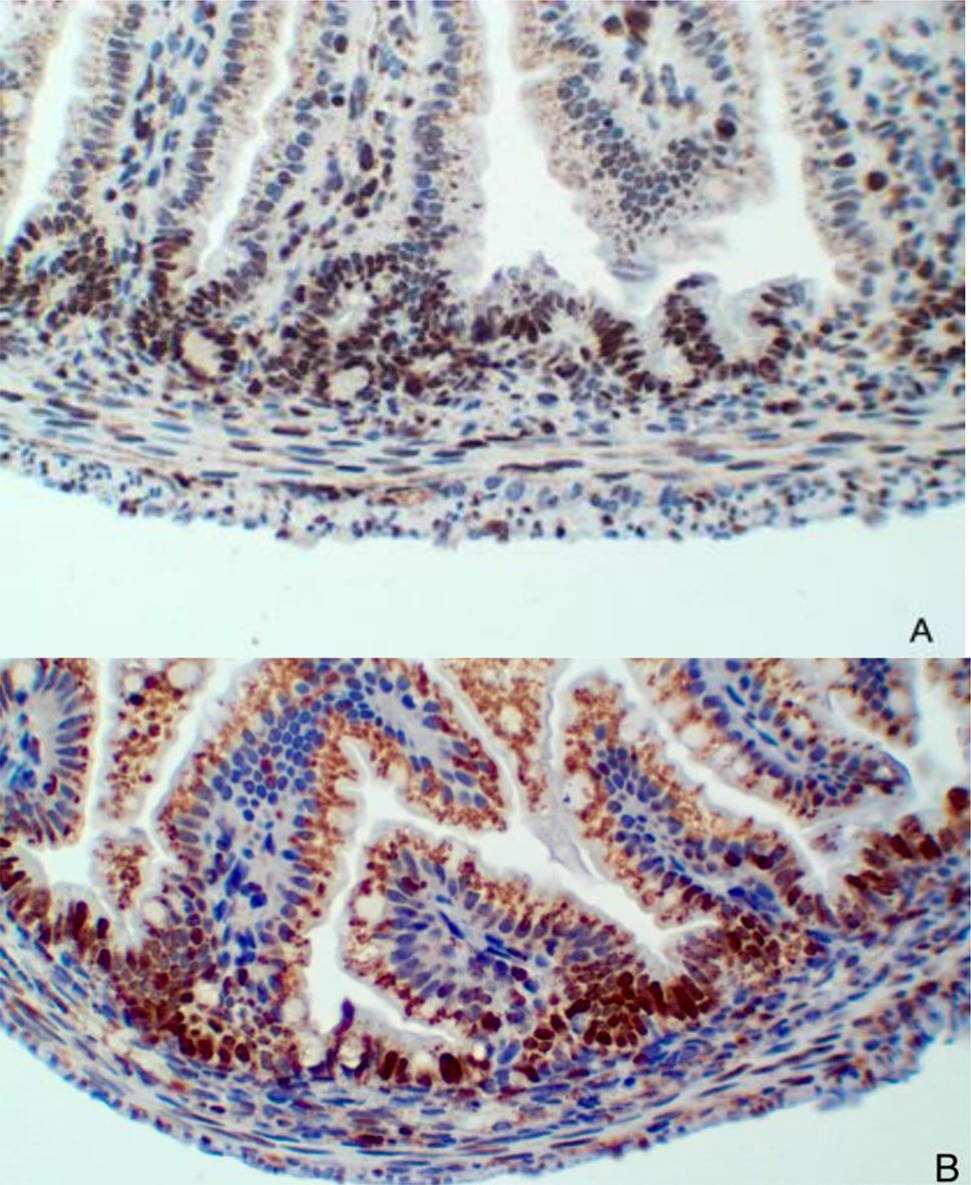
Immunohistochemical analysis of Ki-67. (**A**) Group submitted to the NEC protocol—Reduction of the percentage of brown nuclei cells (Ki-67 stained) in relation to the total number of cells in the proliferative zone of the intestinal mucosa. (**B**) Negative control group—Higher percentage of brown nuclei cells (Ki-67 stained) in relation to the total number of cells in the proliferative zone of the intestinal mucosa.

The group submitted to the NEC protocol obtained a greater reduction in the percentage of cells in the brown nucleus (stained with Ki-67) in relation to the total number of cells in the proliferative zone of the intestinal mucosa, observed in Fig. 6. While the negative control group obtained higher percentage of cells from the brown nucleus (stained with Ki-67) in relation to the total of cells from the proliferative zone of the intestinal mucosa, demonstrating the greater metabolic activity in the intestines of rats not submitted to the NEC protocol.

## Discussion

Formula feeding, hypoxia and hypothermia are considered risk factors for the development of NEC. The mortality rate of the disorder varies from 10 to 50% among newborns treated in neonatal intensive care units^12,13^. In this study, the mortality rate after NEC induction in neonatal rats was 35%, compared with a 38% mortality rate observed by other authors who used the same experimental model^14^.

Contrary to expectations, group C, which received only l-arginine, obtained the highest mortality rate and the lowest survival rate in relation to the other groups. It is difficult to determine which mechanisms are responsible for the harmful effect of l-arginine. We wondered if the substrate supply for NO synthesis, with its consequent increased production, would be the aggressor factor of tissue injury.

A recent study from Alexander’s group defined a dual effect of nitric oxide and therefore arginine. Septic guinea pigs supplemented with low-dose arginine recovered better than controls, while supplemented with high-arginine doses led to catastrophic results^15^. The dose of l-arginine used in this study: was it excessive as a preventive measure and therefore harmful or was it too low to promote the reducing effect on intestinal lesions? In our study, 0.1 mL of 10% l-arginine solution was used intraperitoneally, as in the study from Cintra et al.^10^ However, Cintra et al.^10^ described a protective effect as they show a lower degree of hystologic lesions in the intestine of rats that received l-arginine, contrary to the results found in our study. Our study method differed from the one from Cintra et al.^10^, we did not promote reoxygenation (100% O2) and heating (22 °C) after episodes of hypoxia and hypothermia^16,17^. Maybe reoxygenation and rewarming potentiate the protective action of l-arginine in intestinal lesions of NEC. The hypoxia and hypothermia associated with repeated trauma of intraperitoneal administration of l-arginine possibly leading to bowel perforation.

Group B, which only received sildenafil, had the lowest mortality rate among groups undergoing drug intervention, which was statistically significant. This difference could be due, above all, to the increase in cGMP intracellular levels, which is responsible for stimulating NO production. However, it is noteworthy that group E, submitted to the NEC protocol but not receiving the intervention drug, obtained the highest life span among the NEC groups.Therefore, our study contradicts the current literature by obtaining a higher mortality rate in the NEC groups that received intervention measures with sildenafil and l-arginine. Perhaps the explanation for this fact may be the administered dose of sildenafil and l-arginine, which could have been toxic to newborn rats or the numerous intraperitoneal applications may have caused a very intense endocrine metabolic and immunological response to trauma capable of evolving to death. Could it be that if the doses of sildenafil and l-arginine had lower concentrations or if the intraperitoneal applications were made with an interval greater than 12 h or if the mode of administration of the drugs was different from the intraperitoneal one, the results would be different?

We observed that the final body weight of the groups submitted to the NEC protocol was significantly lower in comparison to the negative control group, which received maternal milk. Other authors have obtained similar results, showing weight loss among animals fed formula milk^12,14^. An exception is a study by Dvorak et al.^12^, that detected an increase in bodyweight in the groups submitted to the NEC protocol.

Regarding the degree of histological injury, the results were similar to those reported in other experimental studies. The NEC induced microscopic changes were such as villous flattening, reduction in the number of villi and thinning of the intestinal wall^18,19^. As shown in Fig. 5, our study did not find villous edema, edema in layers, separation of layers, and peeling of the villi as other studies^12,20^. The probable explanation for this fact would be the immediate fixation of the jejunum and the terminal ileum in 10% buffered formaldehyde, which we performed shortly after the removal of the material. This fixation could have avoided slide artifacts, which could have been considered as injury findings in other studies.

We observed that the intestinal injuries presented high intensity and structural damage in the animals of group E, which were submitted exclusively to the conditions of hypoxia, formula milk, and hypothermia. Hence, the accuracy of the protocol in inducing NEC was confirmed.

The experimental model also demonstrated a higher proliferative index in the groups that received the interventional drugs compared with the positive control group. However, the use of sildenafil and l-arginine has not been able to prevent the development of NEC. One hypothesis is that the intestine of newborns rats would be unable or immature to synthesize NO. Another rationale is that there might not be enough eNOS available nor able to synthesize NO when the supply was high. Sukhotnik and Cols found that exposure of rats to l-arginine does not prevent the intestine from ischemic damage, but reasonably accelerates the repair of the damaged intestinal mucosa^21–23^. Another study showed that l-arginine is not able to inhibit the occurrence of intestinal injuries produced by hypoxia/hypothermia. It has rather shown a higher level of NO and a lower degree of morphological changes in the intestinal wall of animals^10^.

The present study also observed whether there would be any additive effect when concomitantly administering sildenafil and l-arginine to neonatal rats. As verified through the results of group D, we can affirm that there was a reduction in intestinal injury (with a mean of 26.66) and a higher proliferative rate (68.33%) compared with the other groups submitted to the protocol. However, such a reduction was not statistically significant. Therefore, it would not justify the association of substances.

In conclusion, results from the present study demonstrate that the administration of sildenafil and l-arginine is not able to prevent the occurrence of intestinal injuries produced by hypoxia, formula feeding, and hypothermia. Nevertheless, we have evidenced a slightly reduction in the morphological changes in the intestinal wall of animals that received both substances concomitantly, but no statistically significant changes in body weight and intestinal lesion between groups receiving sildenafil and l-arginine combined or individually compared to the NEC control group. Perhaps the time of drug administration was inefficient to observe significant improvement of intestinal lesions, although Cintra et al.^10^ also took a 12-h break between l-arginine administrations made for 3 days, or the NEC induction model was so efficient as to cause a very serious intestinal lesion that could not be prevented by a dose previously tested in other studies. The difference between our study and the experimental model by Jilling et al. and further adapted by Gonçalves et al.^16,17^ it concerns the time of exposure to 100% nitrogen and the administration of the enteral diet. In our study, rats were kept under 100% nitrogen for 70 seconds and fed with 0.1 mL of Esbilac^®^ every 4h, and the experimental model by Jilling et al. and further adapted by Gonçalves et al.^16,17^ left the rats under 60 seconds in an atmosphere of 100% nitrogen and fed with 0.1 mL of Esbilac^®^ every 3 h.

Our study is relevant both for its innovative character of testing sildenafil with l-arginine as a preventive strategy for NEC, as well as for adding new data on l-arginine literature, contradicting previously published studies, which point to these drugs as preventive measures for necrotizing enterocolitis. We were also able to prove the reproducibility of the NEC protocol and its effectiveness in inducing the disease. However, further studies are needed to better understand the highest mortality and lowest survival rate in the group with isolated use of l-arginine. Furthermore, further studies would be interesting to test lower concentrations of sildenafil and l-arginine or to test another route of drug administration to avoid repeated trauma from intraperitoneal applications.

## Methods

### Evaluation by Ethics Committee on animal experimentation

All procedures were approved by the Animal Use Ethics Committee of the Federal University of Paraná (CEUA–UFPR), under number 1249 and verification code 1728778. All methods were performed in accordance with the relevant guidelines and regulations. This study follows the recommendation in the ARRIVE guidelines.

### Experimental model

The experimental NEC model was performed based on the protocol described by Jilling et al. and further adapted by Gonçalves et al.^16,17^. Neonatal rats were fed animal formula milk (Esbilac^®^, PetAg, Hampshire, IL, USA) at 0.1 mL every 4 h for 24 h. Feedings were instituted employing an orogastric tube, adapted from a peripherally inserted central catheter (PICC, 1. 9Fr 26G, 1 lumen, BDs, Sandy, UT, USA). The newborns were placed on a heated surface for the introduction of the orogastric tube at each feeding. Hypoxia was accomplished by stressing the animals with a nitrogen atmosphere (100% N2) for 70 seconds in an anoxic acrylic chamber. Then, neonatal rats were subjected to hypothermia (4 °C for 10 min). This procedure was performed twice a day, at 9 pm and 9 am, for 3 days^24^.

### Experimental design

Forty newborn rats (*Rattus norvegicus*) from the Wistar strain were used. They were divided into five groups:

- Group A—negative control group—5 rats, maternal milk-fed (without NEC).
- Group B—9 rats, fed 0. 1 mL of Esbilac^®^ formula milk every 4 h plus 0.05 mL of intraperitoneal sildenafil (10%) every 12 h.
- Group C—9 rats, fed 0. 1 mL of Esbilac^®^ formula milk every 4 h plus 0.1 mL of intraperitoneal l-arginine (10%) every 12 h.
- Group D—9 rats, fed 0. 1 mL of Esbilac^®^ formula milk every 4 h plus 0.1 mL of intraperitoneal l-arginine (10%) every 12 h plus 0.05 mL of intraperitoneal sildenafil (10%) every 12 h.
- Group E—positive control group—8 rats, fed 0.1 mL of Esbilac^®^ formula milk every 4 h (with NEC) plus 0.1 mL of intraperitoneal saline solution every 12 h.

The neonatal rats in groups B, C, D, and E were removed from maternal contact at 24 h of life. They were placed in a compartmented box under thermal control of 36–38 °C, fed Esbilac^®^ formula milk every 4 h (beginning at 4 pm), and submitted to hypoxia and hypothermia twice a day (9 pm and 9 am) for three days. According to the NEC protocol described by Jilling et al., intestinal damage is progressive and necrotizing enterocolitis sets in after three days of hypoxia and hypothermia events.

Intraperitoneal injections of sildenafil, l-arginine, sildenafil with l-arginine and saline solution were administered every 12 h for 3 days, beginning at 4 pm, 5 h before the start of the NEC induction protocol and maintained until the end of the protocol.

The groups submitted to the NEC protocol (B, C, D, and E) were weighed on all experiment days at 4 pm and immediately before euthanasia. Group A was weighed only once, immediately before euthanasia. The animals of all groups were euthanized at the same time on the fourth day of the experiment, 6 h after the last hypoxia/hypothermia event.

### Sample processing

The experiment was terminated on the fourth day, and animals were euthanized by decapitation. The jejunum was obtained 6 cm from the pylorus, and the terminal ileum was obtained 2 cm from the ileocecal valve. Both organs were removed from each group, then fixed in 10% buffered formaldehyde for histopathological and immunohistochemical analysis.

### Histopathological scoring of slides

Each slide was examined by a blinded observer under an optical binocular microscope. A standard analysis of the slides was performed to assess tissue damage levels, according to the protocol by Dvorak et al. and modified by Granger et al.^25,26^. During the microscopic evaluation, three histological parameters were measured: villous flattening, reduction in the number of villi and thinning of the intestinal wall, of mild, moderate, and severe intensity. A score was assigned for each injury, and an added score was calculated at the end of each investigation. The score ranged from 0 to 36 points. Score 0 represented intact villi, while 36 represented maximum villi injury. The score was calculated as follows: A. no signs of injury: 0—zero; B. villous flattening: mild—2, moderate—4 or severe—6; C. reduction in the number of villi: mild—8, moderate—10 or severe—12; D. thinning of the intestinal wall: mild—14, moderate—16 or severe—18. NEC was considered present in animals with histological scores > 18. Scores were recorded separately, and arbitrary values were assigned according to the signs of injury. Then, an average score was calculated for each group^24^.

### Immunohistochemistry analysis of Ki-67

5-µm-thick section intestinal samples were dewaxed in xylol and rehydrated with alcohol and running water solutions. Antigenic recovery was performed in citrate buffer at 90 °C for 15 min. Endogenous peroxidase was blocked with two baths of hydrogen peroxide for 15 min. Nonspecific protein sites were also blocked according to technical specifications. The primary antibody was the anti-Ki-67 monoclonal antibody, dilution 1: 100, MIB1 clone, refrigerated overnight. Subsequently, the sections were washed and incubated with Polyvalent biotinylated antibody (LSAB kit) and streptavidin—peroxidase (LSAB kit). Washing happened for 30 min at room temperature. After washing, samples were stained with diaminobenzidine (DAB) and counterstained with hematoxylin solution (Mayer’s Protocol). Finally, slides were mounted using an Erv-Mount solution. Positive control was used. Negative control was obtained by replacing the primary antibody, incubating slides in a phosphate-saline buffer. Only active cells with brownish nuclear staining were regarded as positive. The results were evaluated considering the percentage of cells stained in the nuclei in relation to the total number of cells in the proliferative zone of the intestinal mucosa, which is illustrated in Fig. 6. This zone consists of the interface between villi and crypts^27^.

### Statistical analysis

Quantitative variables were expressed as means and standard deviations. Kruskal–Wallis test was used to analyze mortality, survival, body weight, intestinal injury score and Ki-67 proliferation index for independent samples.
